# Expanding clinical profiles and prognostic markers in stiff person syndrome spectrum disorders

**DOI:** 10.1007/s00415-023-12123-0

**Published:** 2023-12-11

**Authors:** Yujie Wang, Chen Hu, Salman Aljarallah, Maria Reyes Mantilla, Loulwah Mukharesh, Alexandra Simpson, Shuvro Roy, Kimystian Harrison, Thomas Shoemaker, Michael Comisac, Alexandra Balshi, Danielle Obando, Daniela A. Pimentel Maldonado, Jacqueline Koshorek, Sarah Snoops, Kathryn C. Fitzgerald, Scott D. Newsome

**Affiliations:** 1 Division of Neuroimmunology and Neurological Infections, Department of Neurology, Johns Hopkins Hospital, Johns Hopkins University School of Medicine, 600 N Wolfe St, Pathology 627, Baltimore, MD 21287 USA; 2 Department of Neurology, University of Washington School of Medicine, Seattle, WA USA; 3 Department of Epidemiology, Johns Hopkins Bloomberg School of Public Health, Baltimore, MD USA

**Keywords:** Stiff person syndrome, Stiff limb syndrome, Progressive encephalomyelitis with rigidity and myoclonus, Anti-GAD65

## Abstract

**Objective:**

To describe the clinical features of a cohort of individuals with stiff person syndrome spectrum disorders (SPSD) and identify potential early predictors of future disability.

**Background:**

There is a need to better understand the full spectrum of clinical and paraclinical features and long-term impact of SPSD.

**Design/Methods:**

Observational study from 1997 to 2022 at Johns Hopkins. Clinical phenotypes included classic SPS, partial SPS (limb or trunk limited), SPS-plus (classic features plus cerebellar/brainstem involvement), and progressive encephalomyelitis with rigidity and myoclonus (PERM). Outcome measures were modified Rankin scale (mRS) and use of assistive device for ambulation. Multivariate logistic regression was used to assess significant predictors of outcomes.

**Results:**

Cohort included 227 individuals with SPSD with mean follow-up of 10 years; 154 classic, 48 SPS-plus, 16 PERM, and 9 partial. Mean age at symptom onset was 42.9 ± 14.1 years, majority were white (69.2%) and female (75.8%). Median time to diagnosis was 36.2 months (longest for SPS-plus and PERM) and 61.2% were initially misdiagnosed. Most had systemic co-morbidities and required assistive devices for ambulation. Female sex (OR 2.08; CI 1.06–4.11) and initial brainstem/cerebellar involvement (OR 4.41; CI 1.63–14.33) predicted worse outcome by mRS. Older age at symptom onset (OR 1.04; CI 1.01–1.06), female sex (OR 1.99; CI 1.01–4.01), Black race (OR 4.14; CI 1.79–10.63), and initial brainstem/cerebellar involvement (OR 2.44; CI 1.04–7.19) predicted worse outcome by use of assistive device. Early implementation of immunotherapy was associated with better outcomes by either mRS (OR 0.45; CI 0.22–0.92) or use of assistive device (OR 0.79; CI 0.66–0.94).

**Conclusions:**

We present the expanding phenotypic variability of this rare spectrum of disorders and highlight potential predictors of future disability.

## Introduction

Stiff person syndrome (SPS) is a rare autoimmune condition that most often is characterized by axial and limb rigidity and spasms and frequently associated with antibodies directed against glutamic acid decarboxylase-65 (GAD65) [1, 2]. These antibodies are not specific to SPS, and occur in other autoimmune diseases, such as type 1 diabetes mellitus and a small subset of the unaffected general population [1–4]. SPS was first described by Moersch and Woltman in 1956 wherein they reported on the clinical features of 14 patients with SPS followed longitudinally at the Mayo Clinic. [5]

The most common phenotype recognized is classic SPS, which comprises muscle spasms and rigidity involving the axial and limb muscles (typically lower extremities) leading to hyperlordosis and gait dysfunction. Additionally, several uncommon phenotypes exist, including involvement of only one limb or torso (partial SPS), symptoms involving predominantly the cerebellum and/or brainstem leading to cerebellar ataxia or ocular motor dysfunction [6], and progressive encephalomyelitis with rigidity and myoclonus (PERM), among others [1–3, 7–9]. With emerging evidence that these rare conditions exist in a clinical phenotypic spectrum, these disorders are better identified as SPS Spectrum Disorders (SPSD) [1, 9–12].

In the last twenty years, there has been increasing literature regarding the clinical presentations and heterogeneity of these disorders [1–3, 7, 11–14]. Given the rarity of these disorders, we aim to add to the body of literature reviewing the clinical profiles of these individuals, to hopefully advance our understanding of the disease presentations, disease course over time and response to treatments and interventions [1–3, 7, 9, 11, 12, 14].

We discuss the demographics, expanding clinical phenotypes, key history and examination features, and immunological profiles, as well as describe potential early predictors of clinical outcomes in individuals affected with these rare disorders. Our major aims are to increase awareness of SPSD and expand the clinical understanding of these interrelated conditions.

## Methods

### Standard protocol approvals, registrations, and patient consents

This study was approved by the Johns Hopkins University Institutional Review Board. All active participants provided written informed consent as part of an ongoing, observational study at Johns Hopkins University. De-identified data that support the findings of this study would be made available to approved researchers via reasonable request to the corresponding author.

### Design, study population, inclusion, and exclusion criteria

This study has both retrospective and prospective components. The retrospective portion included individuals seen at Johns Hopkins University from January 1, 1997 to June 30, 2008, and the prospective portion included individuals seen from July 1, 2008 to July 1, 2022. We included adults (≥ 18 years old) with a diagnosis of SPSD determined by an SPSD expert (S.D.N.) based on clinical presentation, examination findings, paraclinical studies, and lack of a better explanation for signs and symptoms of included individuals. Participants with a history of pure cerebellar ataxia (with no musculoskeletal manifestations), autoimmune epilepsy and primary autoimmune encephalitis were excluded from this study.

A total of 227 individuals were identified. Of these, 211 were seen and followed by S.D.N, while 16 were not seen by S.D.N. but rather a neuromuscular or a movement disorders subspecialist at Johns Hopkins University. Participants were seen at least once every 12 months, and on average had 5.12 clinic visits over the study period.

### Clinical phenotypes, data review, and outcome measures

We divided the participants into specific phenotypes: classic SPS, SPS-plus, PERM, and partial SPS. Classic SPS was defined as patients with torso and/or involvement of extremities (lower > upper) based on previously published criteria [14]. SPS-plus was defined as patients who exhibited some or all classic features along with cerebellar and/or brainstem manifestations. PERM was defined as patients who presented with severe spasm and rigidity with additional features of encephalopathy, myoclonus, brainstem dysfunction, and/or autonomic dysfunction. Partial SPS were defined as stiffness and spasms limited to a specific body region (e.g., stiff leg, stiff trunk) [2, 3, 9, 11]. The clinical phenotype at most recent follow-up was used as the individual’s final phenotype categorization.

Additional clinical data (such as demographics, reported symptoms, co-morbid conditions, examination findings, and ancillary testing results) were either collected retrospectively by review of the available electronic medical records or collected prospectively in a standardized format depending on the time of enrollment of the individual.

As a clinical outcome measure, modified Rankin Scale (mRS, 0 = no symptoms, 1 = no significant disability, 2 = slight disability, 3 = moderate disability, 4 = moderately severe disability, 5 = severe disability, 6 = dead) was calculated by raters blinded to the participants’ clinical phenotype (Y.W., L.M.). Additional clinical outcome measure included ambulation status and device usage (independently ambulatory, use of cane or walking stick, use of bilateral aide, such as a walker, use of a wheelchair, or bedbound) at initial clinic visit as well as last clinic visit. We additionally defined poor outcome as mRS > 2 or use of an assistive device for ambulation at the most recent visit.

### Autoantibody testing

Commercially available autoantibody testing was used as part of standard clinical practice in the care of these individuals. Serum anti-GAD65 levels were measured using either an Enzyme Linked Immunoassay (ELISA) method (Johns Hopkins Laboratories, Baltimore, MD; Quest Diagnostics, Chantilly, VA [expressed in IU/mL]) or utilizing a Radioimmunoassay (RIA) method (Mayo Clinic Laboratories, Rochester, MN [expressed in nmol/mL]). For the ELISA method, values at or above 10,000 IU/mL were designated as high, and for the RIA method, the value was at or above 20 nmol/mL based on previous literature review or as defined directly by the laboratory. In addition, due to varying measurement techniques, normative values, and units of measurement across different clinical laboratories, values were standardized across participants by dividing antibody levels into tertiles (low, middle, and high) with the subsequent creation of a tertile-based variable. For the anti-GAD65 assay in cerebrospinal fluid (CSF), the Mayo Clinic Laboratories was used. For the anti-amphiphysin and -glycine receptor assays, the Mayo Clinic Laboratories was used.

### Statistical analyses

Statistical analyses were performed using R (Version 4.0.3). P-values were reported at the 0.05 significance level. Continuous and categorical variables were reported as mean (± standard deviation) or median (IQR) and number (percentage), respectively. We compared differences across multiple phenotypes by the Kruskal–Wallis, chi-square, or Fisher’s exact test as appropriate.

Associations of individual initial symptom(s) (e.g., spasm/stiffness, brainstem/cerebellar) with each available outcome measure (e.g., mRS > 2, use of assistive device for ambulation) at the last clinical follow-up were separately evaluated by logistic regression models adjusting for age of symptom onset, disease duration, sex, race, titer class of anti-GAD65 and binary variable indicating whether immunotherapy was initiated within 3 years of symptom onset.

## Results

### Demographics

Table 1 outlines the demographic features of the entire cohort (*n* = 227). The majority had classic SPS phenotype (*n* = 154, 67.8%) followed by SPS-plus (*n* = 48, 21.1%). The average age at symptom onset was 42.9 ± 14.1 years. There was a higher female to male ratio (3:1). The majority of participants with SPSD were white (*n* = 157, 69.2%), though the SPS-plus phenotype included a higher proportion of Black participants (*n* = 19, 39.6%) when compared to other phenotypes. Several common co-existing medical co-morbidites were identified including thyroid disease (36.6%), diabetes mellitus (31.7%), and vitamin B12 deficiency (16.3%). There was also a high proportion of patients with depression (30.8%) and/or anxiety (44.9%).

**Table 1:** Overall demographics and most commonly identified co-morbid conditions of individuals with stiff person syndrome spectrum disorders

	Overall (*n* = 227)	Classic SPS (*n* = 154)	SPS Plus (*n* = 48)	PERM (*n* = 16)	Partial SPS (*n* = 9)	*p*-value*
Age at onset, y, mean (SD)	42.9 (14.1)	42.9 (13.6)	45.2 (13.8)	35.4(16.9)	44.8 (18.0)	0.11
Female, *n* (%)	172 (75.8)	117 (76.0)	36 (75.0)	14 (87.5)	5 (55.6)	0.36
Race, *n* (%)						0.21
White	157 (69.2)	118 (76.6)	25 (52.1)	9 (56.2)	5 (55.6)	
Black	50 (22.0)	25 (16.2)	19 (39.6)	5 (31.2)	1 (11.1)	
Asian	5 (2.1)	4 (2.6)	1 (2.1)	0	0	
Other	15 (6.6)	7 (4.5)	3 (6.2)	2 (12.5)	3 (33.3)	
Hispanic ethnicity, *n* (%)	9 (4.0)	7 (4.5)	0	2 (12.5)	0	0.14
Misdiagnosis, *n* (%)	139 (61.2)	92 (59.7)	32 (66.7)	11 (68.8)	4 (44.4)	0.53
Time to diagnosis, months, median (IQR)	36.2 (12.0–79.5)	33.1 (11.3–72.0)	52.0 (11.0–97.5)	53.3 (18.1–81.0)	36.8 (28.0–63.0)	0.07
Time to diagnosis, months, mean (SD)	56.7 (62.9)	54.1 (64.8)	69.3 (69.3)	51.0 (36.5)	45.4 (21.6)	0.10
Duration of follow-up, y, mean (SD)	10.0 (7.5)	9.9 (7.7)	11.3 (8.0)	9.0 (4.6)	7.2 (3.9)	0.39
Most common co-morbid medical conditions						
Diabetes, *n* (%)	72 (31.7)	49 (31.8)	15 (31.2)	3 (18.8)	5 (55.6)	0.50
Thyroid disease, *n* (%)	83 (36.6)	58 (37.7)	16 (33.3)	7 (43.8)	2 (22.2)	1.00
Vitamin B12 deficiency,* n* (%)	37 (16.3)	30 (19.5)	7 (14.6)	0	0	0.27
Vitiligo, *n* (%)	13 (5.7)	8 (5.2)	3 (6.2)	1 (6.2)	1 (11.1)	0.81
SLE, *n* (%)	8 (3.5)	5 (3.2)	2 (4.2)	0	1 (11.1)	0.70
Sjogren’s, *n* (%)	10 (4.4)	7 (4.5)	2 (4.2)	0	1 (11.1)	0.74
Anxiety, *n* (%)	102 (44.9)	72 (46.8)	17 (35.4)	7 (43.8)	6 (66.7)	0.65
Depression, *n* (%)	70 (30.8)	52 (33.8)	12 (25.0)	3 (18.8)	3 (33.3)	0.72
Paraneoplastic syndrome, *n* (%)	9 (3.9)	5 (55.6)	2 (22.2)	0 (0.0)	1(11.1)	0.42

*SPS* = stiff person syndrome, *PERM* progressive encephalomyelitis with rigidity and myoclonus, *SLE* systemic lupus erythematosus, *SD* standard deviation, *IQR* interquartile range, *y* year(s), *n* sample size

*p-values reported are overall difference across all phenotypes

More than half of participants were initially mis-diagnosed with an alternative disorder (*n* = 139, 61.2%). The most common alternative diagnoses included spondyloarthritis/arthropathy, fibromyalgia, unspecified neuropathy, and functional neurologic disorder. These misdiagnoses often resulted in substantial delays in diagnosis as the median time from symptom onset to formal SPSD diagnosis was 36.2 months (IQR 12.0–79.5 months). More specifically, patients with PERM had the longest time from symptom onset to diagnosis (53.3 months [IQR 18.1–81.0 months]) followed by SPS-plus (52.0 months [IQR 11.0–97.5 months]). In patients with an underlying paraneoplastic process (3.9%), the malignancy was identified within 5 years from SPSD symptom onset. The associated autoantibodies were anti-GAD65 (4), anti-amphiphysin (3), anti-GAD65 and -amphiphysin (1), and negative for anti-GAD65 but positive for anti-Hu antibodies (1). The associated cancers were breast cancer (5), small cell lung cancer (2), rectal adenocarcinoma (1), and thymoma (1).

Majority were found to have an elevated anti-GAD65 by one or more of the clinically available laboratory testing methods (*n* = 197, 86.8%), and the antibody titer level was high in most cases (Table 2). A smaller proportion of participants had anti-amphiphysin (n = 6, 2.6%) or anti-glycine receptor (n = 11, 4.8%) identified.

**Table 2:** Immunological profiles of individuals with stiff person syndrome spectrum disorders

	Overall (n = 227)	Classic SPS (n = 154)	SPS Plus (n = 48)	PERM (n = 16)	Partial SPS (n = 9)
Serum Anti-GAD65 + , *n* (%)	197 (86.8)	130 (84.4)	45 (93.8)	15 (93.8)	7 (77.8)
Quest and Hopkins*, *n* (%)	175	114	42	15	4
< 10,000 IU/mL	80 (45.7)	58 (50.9)	12 (28.6)	6 (40.0)	2 (50.0)
> = 10,000 IU/mL	95 (54.3)	56 (49.1)	28 (71.4)	9 (60.0)	2 (50.0)
Mayo Labs^, *n* (%)	29	22	5	1	1
< 20 nmol/L	10 (34.5)	9 (40.9)	0	1 (100)	0
>= 20 nmol/L	19 (65.5)	13 (59.1)	5 (100)	0	1 (100)
Quest and Hopkins*, n (%)	175	114	42	15	4
1st tertile (<117)	27 (15.4)	20 (17.5)	5 (11.9)	1 (6.7)	1 (25.0)
2nd tertile (117–24,576)	61 (34.9)	45 (39.5)	10 (23.8)	5 (33.3)	1 (25.0)
3rd tertile (24,576–2,281,100)	87 (49.7)	49 (43.0)	27 (64.3)	9 (60.0)	2 (50.0)
Mayo Labs^, *n* (%)	29	22	5	1	1
1st tertile (< 44.7)	11 (37.9)	9 (40.9)	0	1 (100)	1 (100)
2nd tertile (44.7–289.7)	8 (27.6)	6 (27.3)	2 (40.0)	0	0
3rd tertile (289.7–1,587)	10 (34.5)	7 (31.8)	3 (60.0)	0	0
CSF anti-GAD65 + , *n* (%)	45/72 (62.5)	27/50 (54.0)	13/14 (92.9)	3/4 (75.0)	2/4 (50.0)
Anti-amphiphysin, *n* (%)	6 (2.6)	6 (3.9)	0	0	0
Anti-glycine receptor, *n* (%)	11 (4.8)	9 (5.8)	1 (2.1)	1 (6.2)	0
CSF restricted oligoclonal banding, *n* (%)	7/99 (7.1)	3/65 (4.6)	1/24 (4.2)	2/6 (33.3)	1/4 (25.0)
CSF elevated IgG index, *n* (%)	8/54 (14.8)	6/36 (16.7)	1/11 (9.1)	0/3	1/4 (25.0)

*SPS* stiff person syndrome, *PERM* progressive encephalomyelitis with rigidity and myoclonus, *GAD* glutamic acid decarboxylase, *CSF* cerebrospinal fluid, *Ig* immunoglobulin, *n* sample size

*Enzyme Linked Immunoassay (ELISA), units International Units (IU)/mL

^Radioimmunoassay (RIA), units nmol/mL.

### Clinical characteristics of specific phenotypes

#### Classic SPS phenotype

**Fig. 1:**
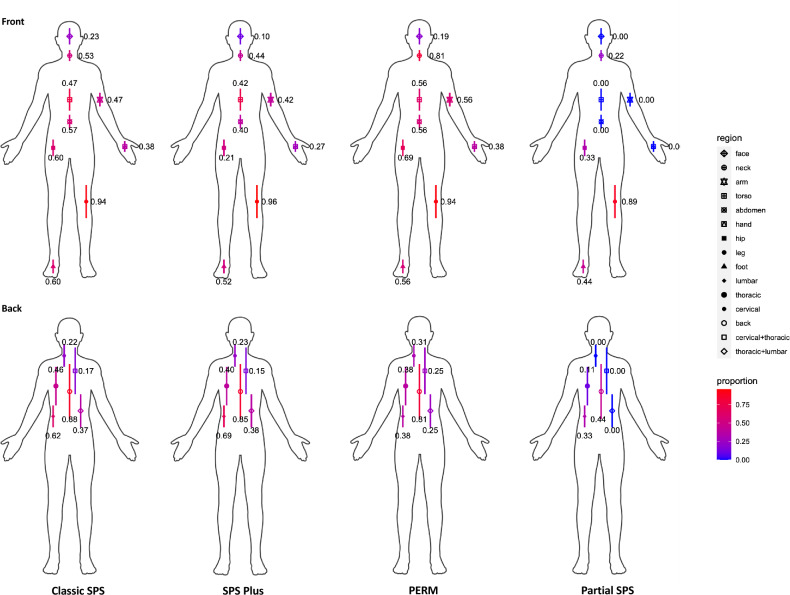
Anatomical involvement of musculoskeletal symptoms among stiff person syndrome spectrum disorders. Locations where participants experienced stiffness and/or spasms based on phenotype. In classic stiff person syndrome (SPS), SPS-plus, and progressive encephalomyelitis with rigidity and myoclonus (PERM) phenotypes, the most commonly affected regions were the axial musculature (torso, back) and lower limbs. PERM had higher proportion of neck/cervical musculature affected. In partial SPS, lower limb was most commonly affected. Color scheme with red indicating higher and blue indicating lower proportion affected

Figure 1 demonstrates the anatomical involvement of the musculoskeletal symptoms by phenotype. In the classic SPS phenotype, the axial musculature and the lower extremities were most commonly affected by stiffness and/or spasms. Table 3 describes other important clinical characteristics seen in classic SPS including hyperlordosis (46.8%), hypertonia/rigidity (85.1%) and hyperreflexia (70.1%), among other findings. Hypersensitivity triggers and related symptoms, such as agoraphobia, heightened/exaggerated startle reflex, and others, were common. Other less reported symptoms included photosensitivity, cognitive or mood dysregulation, dyspnea, chest pain or tightness, and gastrointestinal symptoms. The majority required an assistive device at last follow-up (*n* = 106, 68.8%).

**Table 3:** Most common pertinent clinical/examination characteristics/findings of stiff person syndrome spectrum disorders

	Overall (*n* = 227)	Classic SPS (*n* = 154)	SPS Plus (*n* = 48)	PERM (*n* = 16)	Partial SPS (*n* = 9)
Axial stiffness and spasms, *n* (%)	212 (92.5)	147 (95.5)	44 (91.7)	14 (87.5)	5 (55.6)
Appendicular stiffness and spasms, *n* (%)	217 (95.6)	148 (96.1)	46 (95.8)	15 (93.8)	8 (88.9)
Hyperlordosis, *n* (%)	106 (46.7)	72 (46.8)	25 (52.1)	7 (43.8)	1 (11.1)
Hyperreflexia, *n* (%)	165 (72.7)	108 (70.1)	34 (70.8)	12 (75.0)	7 (77.8)
Hypertonia, *n* (%)	196 (86.3)	131 (85.1)	41 (91.1)	14 (87.5)	6 (66.7)
Incoordination, *n* (%)	121 (51.5)	63 (40.9)	36 (80.0)	10 (62.5)	3 (33.3)
Diplopia, *n* (%)	54 (23.8)	17 (11.0)	31 (64.6)	5 (31.2)	1 (11.1)
Vertigo, *n* (%)	60 (26.4)	26 (16.9)	26 (54.2)	7 (43.8)	1 (11.1)
Ocular motor dysfunction, *n* (%)	64 (28.2)	15 (9.7)	35 (72.9)	13 (81.3)	1 (11.1)
Photosensitivity, *n* (%)	57 (25.1)	30 (19.5)	20 (41.7)	6 (37.5)	1 (11.1)
Cognitive symptoms, *n* (%)	102 (44.9)	71 (46.1)	19 (39.6)	9 (56.2)	3 (33.3)
Need for ambulation device use, *n* (%)	164 (72.2)	106 (68.8)	39 (79.2)	15 (93.8)	7 (77.8)
Unilateral support	52 (22.9)	33 (21.4)	17 (37.8)	2 (12.5)	0
Bilateral support	66 (29.1)	41 (26.6)	14 (29.2)	6 (37.5)	5 (55.6)
Wheelchair	25 (11.0)	9 (5.8)	8 (16.7)	7 (43.8)	1 (11.1)
Unspecified support device	7 (3.1)	23 (14.9)	0	0	1 (11.1)
Hypersensitivity triggers, *n* (%)					
Agoraphobia	87 (38.3)	59 (38.3)	18 (37.5)	9 (56.2)	1 (11.1)
Startle	130 (57.3)	90 (58.4)	26 (54.2)	11 (68.8)	3 (33.3)
Cold	129 (56.8)	90 (58.4)	25 (52.1)	10 (62.5)	4 (44.4)
Open space	84 (37.0)	62 (40.3)	15 (31.2)	6 (37.5)	1 (11.1)
Stress	168 (74.0)	118 (76.6)	32 (66.7)	13 (81.2)	5 (55.6)
Noise	141 (62.1)	100 (64.9)		11 (68.8)	3 (33.3)
EMG findings of co-contraction, continuous motor unit activity, *n* (%)	37/158 (23.4)	27/113 (23.9)	5/29 (17.2)	3/10 (30.0)	2/5 (40.0)

*SPS* stiff person syndrome, *PERM* progressive encephalomyelitis with rigidity and myoclonus, *n* sample size, *EMG* electromyography

Included symptoms/signs were based on frequency of occurrence being more than 20% of entire cohort.

#### SPS-plus phenotype

The anatomical involvement of the musculoskeletal symptoms in SPS-plus phenotype was similar to what was seen in the classic SPS phenotype (Fig. 1) as were other clinical characteristics as delineated in Table 3. However, based on phenotype definition, SPS-plus was noted to have a high percentage of patients with ocular motor and/or cerebellar signs and symptoms (e.g., diplopia, oculomotor paresis, nystagmus, incoordination, vertigo, etc.). Hypersensitivity triggers and related symptoms were also commonly present. Other associated symptoms that were reported include cognitive or mood dysregulation, and less commonly dysphagia, dyspnea, chest pain or tightness, and gastrointestinal symptoms. Wheelchair use (16.7%) was higher in this group compared to the classic SPS phenotype, and assistive device use was common (*n* = 39, 79.2%).

#### Progressive encephalomyelitis with rigidity and myoclonus

The pattern of musculoskeletal symptoms in PERM was similar to what was found in the classic SPS and SPS-plus phenotypes, though those with PERM had higher percentage of involvement of the neck/cervical musculature (Fig. 1). Other associated signs and symptoms that were more prevalent in PERM compared with the other phenotypes included ocular motor dysfunction (81.3%), cognitive dysfunction (56.2%), and startle response (68.8%) (Table 3). Wheelchair use was high (43.8%) in this group, and when they were ambulatory, more commonly a walker or bilateral support system was used (37.5%).

#### Partial SPS

Musculoskeletal involvement was predominately of the lower extremities (Fig. 1) in individuals who presented with partial SPS, such as stiff limb syndrome. Individuals reported less of the atypical symptoms than other phenotypes.

### Paraclinical profiles of patients

The most commonly identified associated autoantibody was anti-GAD65 (86.8%). In the majority of individuals, the value was high based on previously discussed clinical laboratory determined values (see methods section). A minority of individuals had the less commonly associated autoantibodies, anti-amphiphysin (*n* = 6, 2.6%) and -glycine receptor (*n* = 11, 4.8%). The individuals with anti-amphiphysin identified mostly presented with classic SPS phenotype, and all but one was paraneoplastic associated with breast cancer. The individuals with anti-glycine receptor were seen in all the phenotypes. As these autoantibodies were obtained on clinical-based, not all participants had all autoantibodies tested.

CSF, when obtained, identified anti-GAD65 in 62.5% of individuals. CSF restricted oligoclonal banding and/or abnormal immunoglobulin G synthesis were identified less commonly (7.1% and 14.8%, respectively).

Continuous motor unit activity and/or co-contraction of agonist and antagonist muscles were noted to occur in 37 out of 158 (23.4%) patients who underwent an electromyography (EMG) test. The low percentage of positive EMGs could have been influenced by SPSD treatments (e.g., benzodiazepines) since many patients were on these treatments at the time of their EMG.

### Immune and symptomatic interventions

#### Immune-based treatments

A total of 180 patients (79.3%) used at least one immune-based treatment at some point in their treatment course, with 96 (42.3%) using combination (two or more) immune-based treatments. The most commonly used immunotherapy was intravenous immunoglobulin (IVIg) (*n* = 175, 77.1%), followed by rituximab (*n* = 62, 27.3%), plasmapheresis/plasma exchange (*n* = 52, 22.9%), and steroids (*n* = 36, 15.9%). In addition, four individuals underwent autologous hematopoietic stem cell transplant therapy at outside facilities.

The mean time from symptom onset to first immune therapy used was 55.9 ± 62.6 months. The most common reasons for discontinuation of immune therapy were lack of efficacy and/or side effects of therapy.

#### Symptomatic pharmacological and non-pharmacologic treatments

The majority of patients used a combination of therapies to help mitigate symptoms (> 75%). The most common oral symptomatic medications used were GABAergic agonists; benzodiazepines (diazepam and clonazepam being the most common), baclofen, and gabapentin. Other relevant oral pharmacological medications included tizanidine, methocarbamol, and pregabalin. Forty-three individuals underwent botulinum toxin injections, of which 58.1% reported it to be beneficial for their musculoskeletal symptoms. Medications with norepinephrine reuptake inhibitor (NRI) mechanism of action were temporally associated with worsening symptoms, hence, avoided in most patients once this association became apparent [15].

The majority of individuals participated in some form of non-pharmacologic therapies/treatments and found them to be beneficial for their symptoms, overall function, and quality of life. These therapies/treatments were diverse including most commonly physical and occupational therapy, cognitive/behavioral therapies, acupuncture and acupressure, massage therapy, chiropractic and osteopathic manipulation, yoga and meditation, heat and ultrasound therapy, and aquatherapy.

### Association with future level of disability in SPSD

Female sex (ref: male, OR 2.08, 95% Confidence Interval [CI] 1.06–4.11), initial presenting symptoms of cerebellar and/or brainstem involvement (ref: presenting with stiffness/spasms, OR 4.41, 95% CI 1.63–14.33), and unexposed to immune therapy within 3 years of symptom onset (OR 2.22, 95% CI 1.09–4.55) were associated with worse level of disability as measured by mRS. Female sex (ref: male, OR 1.99, 95% CI 1.01–4.01), Black race (ref: White, OR 4.14, 95% CI 1.79–10.63), older age of symptom onset (OR 1.04, 95% CI 1.01–1.06), initial presenting symptoms of cerebellar and/or brainstem involvement (ref: presenting with stiffness/spasms, OR 2.44, 95% CI 1.04–7.19), and unexposed to immune therapy within 3 years of symptom onset (OR 1.27, 95% CI 1.06–1.52) were associated with worse level of disability as measured by the use of assistive device for ambulation. High anti-GAD65 titer level in serum was not predictive of worse clinical outcomes. Full data are shown in Tables 4 and 5.

**Table 4:** Multi-variate logistic regression model demonstrates that female sex and initial symptoms of brainstem or cerebellar pathology predict poorer outcome as measured by modified Rankin scale > 2 at last follow-up

	Adjusted OR (95% CI)	*p*-value
Age at symptom onset, years	1.01 (0.99–1.03)	0.36
Sex – Female	2.08 (1.06–4.11)	0.03
Race – Black/African American	2.11 (1.00–4.69)	0.06
Disease duration	1.05 (1.00–1.11)	0.09
Initial symptoms		
Stiffness/spasms	0.69 (0.37–1.26)	0.23
Cerebellar/Brainstem	4.41 (1.63–14.33)	0.006
High-titer GAD antibody (defined as ELISA > 10,000 IU or RIA > 20 nmol)	1.59 (0.74–3.41)	0.23
Immunotherapy started within 2–3 years	0.45 (0.22, 0.92)	0.03
Unexposed to immunotherapy within 3 years of symptom onset	2.22 (1.09, 4.55)	0.03

*GAD* glutamic acid decarboxylase, *ELISA* Enzyme-Linked Immunoassay, *IU* units International Units/mL, *RIA* radioimmunoassay, *mL* units nmol/milliliter

**Table 5:** Multi-variate logistic regression model demonstrates that older age at symptom onset, female sex, Black/African American race, and initial symptoms of brainstem or cerebellar pathology predict poorer outcome as measured by the dependence on ambulatory assistive device at last follow-up

	Adjusted OR (95% CI)	*p*-value
Age at symptom onset, years	1.04 (1.01–1.06)	0.002
Sex—Female	1.99 (1.01–4.01)	0.05
Race – Black/African American	4.14 (1.79–10.63)	0.002
Disease duration	1.04 (0.99–1.09)	0.17
Initial symptoms		
Stiffness/spasms	1.12 (0.60–2.11)	0.71
Cerebellar/Brainstem	2.44 (1.04–7.19)	0.04
High-titer GAD antibody (defined as ELISA > 10,000 IU or RIA > 20 nmol)	1.27 (0.57–2.80)	0.56
Immunotherapy started within 2–3 years	0.79 (0.66, 0.94)	0.007
Unexposed to immunotherapy within 3 years of symptom onset	1.27 (1.06, 1.52)	0.007

*GAD* glutamic acid decarboxylase, *ELISA* enzyme Linked Immunoassay, units International Units (IU)/mL, *RIA* Radioimmunoassay, *mL* units nmol/milliliter

## Discussion

This study reinforces the phenotypic heterogeneity of SPSD [1, 2, 7, 9, 12] and supports the expanding spectrum of non-classical features and reports potential early clinical predictors of future disability in SPSD.

In terms of the phenotypic presentation of individuals with SPSD, the anatomic involvement of the musculoskeletal symptomatology was similar across the various phenotypes, demonstrating higher incidence of involvement of axial (particularly trunk for SPS and SPS-plus, and neck/cervical region for PERM) and lower extremity musculature. When the presentation was partial SPS, there was greater involvement of the lower limb(s). Key examination features for those with the predominant musculoskeletal presentations were stiffness or spasms of axial musculature and/or involved limbs, long tract signs, hypertonia (either spasticity or rigidity), and hyperlordosis of the spine. For the rarer phenotypes, there were other features, most typically localizing to brainstem and/or cerebellar dysfunction, such as ataxia, diplopia, nystagmus, and oscillopsia. Such presentations are important to recognize as either a presenting feature of or as a part of SPSD [6, 16–18]. It is important to identify these non-classical presentations given the longer delay in diagnosis for some of these phenotypes (e.g., SPS-plus and PERM) and greater level of future disability as outlined below.

Hypersensitivity triggers were reported in most patients with SPSD (though to a lesser degree than those who were partial SPS phenotype). These core clinical features are important to recognize in the diagnosis of SPSD especially since they can result in the most disabling and isolating aspects of a patient’s disease (e.g., agoraphobia). Moreover, the presence of such features could help aide in an earlier diagnosis and be monitored for treatment over time. In fact, given how common these triggers are in SPSD, Dalakas and colleagues used a heightened sensitivity score as an outcome measure in SPS clinical trials which incorporates some of these common hypersensitivity triggers. Along the same lines, use of increased exteroceptive reflex on EMG may assist as well in diagnosis [19]. In addition, there were other associated symptoms that have been less well recognized, but still frequently reported and identified, including gastrointestinal symptoms [20], cognitive dysfunction [21], mood disorders, and even photosensitivity [22]. These less recognized symptoms are interesting and may suggest SPSD as a more multi-system disorder. It is important to identify these under-recognized symptoms of SPSD to allow for monitoring of disease and treatment of associated symptoms. There is some literature suggesting high incidence of and potentially an increased risk of psychiatric comorbidities in SPS [21, 23, 24]. Recognizing the frequent confounding factors of psychiatric co-morbidities is important as to not mis-attribute such symptoms to psychiatric or psychogenic processes [23, 25, 26] and also to improve the identification of these co-morbid conditions which need treatment.

There are frequent mis-diagnoses as well as delay in diagnosis of SPSD, thus recognizing the salient features of the history and examination are important for timely and accurate diagnosis [14, 23]. This is especially true for patients who present with non-classical features (e.g., median time for diagnosis of PERM was 53.3 months vs. classic SPS was 33.1 months). On the other hand, misdiagnosis and/or over-diagnosis of SPSD is also problematic. Future work, including development of international consensus-based diagnostic criteria, may help aide in improving SPSD diagnosis.

There is frequent co-occurrence of other disorders, many presumed to be autoimmune in nature, most commonly insulin-dependent diabetes mellitus, thyroid disorder (such as thyroiditis), and pernicious anemia [1, 2, 7, 14]. Given the frequency of these conditions, it is recommended to screen for and continue to monitor for their development over time. Consider obtaining a set of specific labs (e.g., HgA1c, thyroid function tests, thyroid antibodies, vitamin B12, methylmalonic acid) on follow-up visits in order to monitor for the development of these co-morbidities.

In terms of treatments, the majority of individuals required a multi-disciplinary approach. This included combinations of symptomatic and immune-based treatments. Benzodiazepines and other medications targeting the GABA-ergic system were used in a majority of individuals with beneficial response, supporting the use of these symptomatic treatments as first-line in SPSD with musculoskeletal presentations [3, 4, 7, 9]. However, medications that inhibit the reuptake of synaptic norepinephrine (e.g., duloxetine, amitriptyline, bupropion hydrochloride, etc.) appeared to worsen musculoskeletal symptoms expanding on a previously published small case series from our group [15]. This recurrent association has prompted some clinicians to recommend avoiding such medications in patients with SPSD.

Though a variety of immunotherapies were used, the most common by far was IVIg, which is likely attributed to previous clinical trial data and retrospective as well as anecdotal data supporting its benefit in SPSD [27–29]. A recent study by Yi and Dalakas demonstrated in a cohort of 36 SPSD patients followed longitudinally, that IVIg reduces symptoms and improved functioning of the majority of patients for at least a few years and some up to several years [28]. This study highlighted the longer-term durability of IVIg treatment in a real-world setting. Based on clinical trial, real-world and anecdotal evidence, higher monthly dosing of IVIg (i.e., 2 grams/kg total) appears to be the most beneficial, although, over time, this can be tailored at the individual level depending on clinical response, tolerability, and other factors [9, 27–29]. The second most common immunotherapy used was rituximab. Though a prior small clinical trial assessing the use of rituximab in SPS yielded disappointing results [30], some individuals appear to respond favorably to rituximab when used for a longer duration [31]. Additionally, immune-based treatments may not improve function in many people with SPSD but help prevent progression (worsening function) in their disease which might be a more realistic treatment goal currently. More robust, prospective studies with longer follow-up duration on rituximab use and other immune-based treatments in SPSD are needed. Many individuals utilized various combinations of non-pharmacological interventions, such as rehabilitation therapies, pain management, and cognitive/behavioral therapies. These non-pharmacological interventions have not been evaluated in a robust fashion in SPSD, however, further study and consideration of inclusion in the multi-modal treatment strategy of these individuals is warranted.

There were potential factors associated with worse long-term outcomes. From a phenotypic perspective, cerebellar and/or brainstem initial presentations were associated with worsening level of disability. This finding further supports a recent study of anti-GAD65 neurological autoimmunity from the Mayo Clinic [1], which identified that cerebellar ataxia predicted poor outcome. This raises the question of timing and initiation of treatments such as immunotherapies in improving these outcomes. From a demographic perspective, female sex, older age, and Black race were associated with worse disability. Currently, there is limited literature regarding race factors in SPSD, though there was a prior study that evaluated 22 Black American and 18 white American individuals with anti-GAD65 neuro-immunity (mainly epilepsy syndromes) that showed that the age of disease onset was lower in Black Americans and the incidence of refractory seizures was higher in Black Americans, hinting that possibly the disease was more aggressive in Black Americans compared to white Americans in their cohort [32]. The underlying reasons to these identified demographic factors contributing to worse disability remain unknown. Demographic factors should be explored in future studies as they may help to identify individuals who are more at-risk for worsened outcomes and for whom certain types of treatments (and timing) may be beneficial to improve long-term outcomes. There may also be consideration of healthcare access, particularly to specialty clinics, for marginalized individuals, that warrants future study.

The current study also showed that earlier initiation of immunotherapy is associated with either stabilization or improvement in the clinical course of SPSD. Other studies have shown immunotherapy in general are associated with better outcomes. For example, positive responses to immunotherapy were shown in a recent survey study from Japan which reviewed 29 individuals with anti-GAD65 SPS and in a single center cohort study from Detroit which reviewed 23 individuals with SPS  [33, 34]. However, currently, there is no consensus on the approach to the timing of immunotherapy [9]. Although, growing literature (including the aforementioned studies) does support the use of immunotherapy in SPSD, particularly IVIg. This includes clinical trial data of IVIg benefit in SPS [27, 29], retrospective cohort data of individuals with SPS treated with IVIg [1, 28], and data from the US Veterans Affairs Health Administration records [35]. There are less robust data on the use of other types of immunotherapies in SPSD [3, 9]. However, there are some case series and studies that provide supportive evidence for the use of corticosteroids, subcutaneous immunoglobulins (SCIg) [36, 37], plasmapheresis/plasma exchange [1, 38–40], rituximab [1, 31, 41], cyclophosphamide [1], and even possibly (though controversially) autologous hematopoietic stem cell transplantation [42, 43]. The treatment approach to individuals with SPSD is more varied in practice [9]. Additionally, there is literature supporting that SPS—in the absence of immunotherapy—is a progressive disorder that leads to accumulating physical disability over time [14, 44]. Therefore, if the goal were to be the stabilization or prevention of such disease progression, the role of immunotherapies and the timing of initiation in achieving this goal should be better elucidated [9]. Early initiation of immunotherapy after symptom onset versus later has the potential to prevent long-term disability as shown in the current study. However, further studies are needed in order to replicate these findings.

There are limitations to this study. Some of the data collected were retrospective in nature, although these data were on a smaller subset of our entire cohort, the majority of patients were followed prospectively. There were smaller sample sizes for the rarest phenotypes studied, which is expected given the limitations of sample sizes in general with rare conditions. The laboratory testing for anti-GAD65 was not standardized within one laboratory but utilized clinically available laboratories at the time, and there is currently a lack of data on how to compare one laboratory’s titers to another when different methodologies are used. However, despite the heterogeneity of clinical laboratories utilized, this is applicable to clinical practice as varied laboratories and methodologies are used for the diagnosis and monitoring of individuals. Future studies comparing the test methodologies and assays are clearly needed. The clinical outcome measures utilized are generic rather than disease-specific. There is a need for more nuanced disease-specific outcome measures for long-term monitoring of disease progression as well as assessing response to various interventions including immunotherapies in SPSD. With regard to treatments utilized, there may be confounding by indication such as earlier initiation of immunotherapies or other treatments in certain individuals.

Despite these limitations, our study adds to the increasing literature on the varied phenotypic presentations of SPSD and identifies potential early clinical predictors of future disability in SPSD. Future work, particularly collaborative and consensus-based approaches, is needed in order to better understand the full impact of SPSD on patients including rarer subtypes and provide improved evidence for the monitoring and treatment of these individuals.
